# Factors Associated With Metabolic Syndrome in a Mediterranean Population: Role of Caffeinated Beverages

**DOI:** 10.2188/jea.JE20130166

**Published:** 2014-07-05

**Authors:** Giuseppe Grosso, Stefano Marventano, Fabio Galvano, Andrzej Pajak, Antonio Mistretta

**Affiliations:** 1 Department of G.F. Ingrassia, Section of Hygiene and Public Health, University of Catania, Catania, Italy; 2 Department of Drug Sciences, Section of Biochemistry, University of Catania, Catania, Italy; 3 Department of Epidemiology and Population Studies, Institute of Public Health, Faculty of Medical Care, Jagiellonian University Medical College, Krakow, Poland

**Keywords:** coffee, tea, polyphenols, caffeine, metabolic syndrome, cardiovascular risk factors

## Abstract

**Background:**

Intake of caffeinated beverages, such as coffee and tea, has been related to improvements in components of metabolic syndrome (MetS), but studies conducted in the Mediterranean region are scarce. The aim of this study was to evaluate whether or not consumption of a variety of beverages containing caffeine was associated with components of MetS in an Italian population.

**Methods:**

From May 2009 to December 2010, a cross-sectional survey was conducted on 1889 inhabitants living in Sicily, southern Italy. Data regarding demographic characteristics, habitual beverage intake, and adherence to the Mediterranean diet were collected, and clinical information was retrieved from the general practitioners’ computer records.

**Results:**

After adjusting for all covariates, coffee (odds ratio [OR] 0.43, 95% confidence interval [CI] 0.27–0.70) and tea (OR 0.51, 95% CI 0.34–0.78) were associated with MetS, whereas no association was observed between caffeine intake and MetS. Among other factors, age, body mass index, physical activity, current smoking, and adherence to Mediterranean diet were associated with having MetS. Triglycerides were inversely associated with consumption of both espresso coffee and tea. The healthy effects of such beverages were more evident in individuals with unhealthy dietary habits.

**Conclusions:**

Although no direct association between caffeine intake and MetS or its components was observed, coffee and tea consumption was significantly related to reduced odds of MetS.

## INTRODUCTION

Metabolic syndrome (MetS) is a common affliction characterized by a constellation of metabolic disorders identified as risk factors for the development of cardiovascular disease (CVD), such as central obesity, impaired glucose tolerance, dyslipidemia, and hypertension.^1^^,^^2^ The prevalence of MetS in the Mediterranean regions is estimated to be 15%–20% in men and 19%–25% in women,^3^^,^^4^ with an increase to as much as 50% of subjects over 70 years old in certain countries.^5^ These rates have further risen during last decade, reaching alarming rates with significant differences between regions due to unhealthy lifestyle and dietary habits. A variety of factors related to the diet have been associated with an increased risk of MetS, including high consumption of refined grains and meat products,^6^ whereas a diet rich in vegetables and fruit is believed to be protective.^7^

In recent years, many authors have turned their attention to coffee and tea consumption and their effects on health.^8^ The beneficial effects of both beverages has been attributed to their high content of antioxidant compounds, such as polyphenols,^9^ and of caffeine, which has been hypothesized to induce metabolism activation leading to lipid mobilization and fat oxidation.^10^^,^^11^ However, the exact mechanisms of these favorable effects remain unclear. Although observational data suggest a benefit, studies specifically exploring the association between coffee and tea consumption and MetS are scarce.^12^^–^^15^ Some studies conducted in Japanese populations have demonstrated a significant inverse correlation between coffee intake and MetS,^13^^,^^14^ whereas studies conducted in European countries failed to demonstrate such a protective effect.^12^^,^^15^ However, no studies exploring the association between coffee intake and MetS have been conducted in the Mediterranean area.

The aim of this study was to evaluate whether or not consumption of a variety of beverages containing caffeine was independently associated with components of MetS in a population living in Italy. Specifically, we tested whether or not the potential association depended on type of beverage or caffeine content.

## METHODS

### Study population

From May 2009 to December 2010, 3254 inhabitants living in Sicily, southern Italy, were selected to enroll in a study conducted on dietary habits and risk of CVD. Details of sampling methodology are described elsewhere.^16^ Briefly, the sampling was performed through the selection of a random pool of 14 general practitioners (GPs) working in the 10 districts of the city of Catania and in the village of Lentini, southern Italy. Of the initial selection, the present study was conducted in a subgroup of 1889 subjects selected according to the availability of data regarding blood samples in the GPs’ computer records. The 2 groups did not substantially differ in background characteristics (mean age: 51.8 and 50.2 years; females: 58.1% and 59.8% of selected and excluded subjects, respectively) or variables of interest concerning health status (obesity: 13.9% and 13.7%; hypertension: 55.3% and 56.3%; diabetes: 5.7% and 6.6% of selected and excluded subjects, respectively). Before the interview, participants were informed about the procedures of the study, and an informed consent form was collected. The retrieved data were confidential, and the study followed the ethical consideration provided by the ethics committee of the University of Catania.

### Data collection

Trained interviewers collected all required information using a standardized procedure. The survey included basic demographic items, such as age, gender, occupation, and education level, categorized as displayed in Table 1. Information regarding current smoking, alcohol drinking (>12 g/day), and physical activity level were also collected as described elsewhere.^16^ Dietary information was collected through a validated questionnaire^17^ and included questions regarding usual drinking of beverages containing caffeine, specifically frequency (ie, how many times per week/month), type (ie, espresso coffee, instant coffee, tea, soft and energy drinks, and chocolate), and quantity (ie, 35-mL cups/day of espresso coffee or 150-mL cups/day of tea). The amount of caffeine was computed by a web program containing the food composition tables of the European Institute of Oncology (EIO) database.^18^ The MedDietScore (range: 0–55) developed by Panagiotakos et al^19^ was used to assess adherence to the Mediterranean diet (MD). We divided the score into tertiles, with higher tertile scores indicating greater adherence to the dietary pattern.

**Table 1.** Main characteristics of the study population by metabolic syndrome (*N* = 1889)

	Total (*n* = 1889)	Metabolic syndrome		

		No	Yes	*P*-value
Gender, *n* (%)				0.99
Male	760 (40.2)	669 (40.2)	91 (40.3)	
Female	1129 (59.8)	994 (59.8)	135 (59.7)	
Age, years, mean (SD)	50.2 (16.3)	49.4 (16.3)	55.2 (14.7)	<0.001
BMI, kg/m^2^, mean (SD)	25.6 (3.8)	25.1 (3.6)	29.0 (3.2)	<0.001
Educational level, *n* (%)				<0.05
Low	841 (44.5)	720 (43.3)	121 (53.5)	
Medium	892 (47.2)	796 (47.9)	96 (42.5)	
High	156 (8.3)	147 (8.8)	9 (4.0)	
Socio-economic status, *n* (%)				0.64
Low	868 (46)	763 (45.9)	105 (46.5)	
Medium	749 (39.7)	656 (39.4)	93 (41.2)	
High	272 (14.4)	244 (14.7)	28 (12.4)	
Energy intake (kcal/day), mean (SD)	1965 (642)	1950 (627)	2069 (735)	<0.05
Current smoking, *n* (%)	489 (25.9)	411 (24.7)	78 (34.5)	<0.05
Alcohol drinking, *n* (%)	652 (34.5)	573 (34.5)	79 (35.0)	0.88
Physical activity, *n* (%)				<0.001
Low	916 (48.5)	797 (47.9)	119 (52.6)	
Moderate	673 (35.6)	580 (34.9)	93 (41.2)	
High	300 (15.9)	286 (17.2)	14 (6.2)	
MedDietScore, *n* (%)				<0.001
Low	413 (21.9)	339 (20.4)	74 (32.7)	
Medium	1234 (65.3)	1095 (65.8)	139 (61.5)	
High	242 (12.8)	229 (13.8)	13 (5.8)	
Caffeine (mg/day), *n* (%)				<0.05
<80 mg	590 (31.2)	497 (29.9)	93 (41.2)	
80–160 mg	847 (44.8)	767 (46.1)	80 (35.4)	
>160 mg	452 (23.9)	399 (24.0)	53 (23.5)	
Source of caffeine (≥1/day), *n* (%)				
Soft Drinks	1005 (53.2)	881 (53)	124 (54.9)	0.89
Tea	623 (33.0)	566 (34.0)	57 (25.2)	<0.05
Energy drinks	184 (9.7)	165 (9.9)	19 (8.4)	0.47
Coffee	892 (47.2)	805 (48.4)	87 (38.5)	<0.05
Type of coffee, *n* (%)				
Decaffeinated	66 (3.5)	56 (3.4)	10 (4.4)	0.42
Instant	87 (4.6)	78 (4.7)	9 (4.0)	0.63
Espresso	816 (43.2)	737 (44.3)	79 (35.0)	<0.05

BMI, body mass index; SD, standard deviation.

Weight and height were measured to obtain body mass index (BMI; kg/m^2^). Obesity was defined as a BMI ≥ 30 kg/m^2^. Waist circumference (WC; cm) was measured at the level of midway between the 12th rib and the iliac crest. Participants’ blood pressure levels were measured at the end of the physical examination with subject in sitting position and after at least 30 minutes at rest. Blood pressure measurements were taken twice at the right arm relaxed and well supported by a table, at an angle of 45° from the trunk. Blood lipids (total, HDL-cholesterol, LDL-cholesterol, and triglycerides) and fasting plasma glucose levels recorded within 6 months from the visit were retrieved from the GPs’ computer records.

MetS was defined according to the International Diabetes Federation definition,^20^ namely having central obesity (waist circumference ≥90 cm in men and ≥80 cm in women) and any two of the following: (i) triglycerides >150 mg/dL (1.7 mmol/L), or specific treatment for this lipid abnormality; (ii) HDL cholesterol <40 mg/dL (1.03 mmol/L) in males, <50 mg/dL (1.29 mmol/L) in females, or specific treatment for this lipid abnormality; (iii) systolic blood pressure >130 mm Hg or diastolic blood pressure >85 mm Hg, or treatment of previously diagnosed hypertension; and (iv) fasting plasma glucose >100 mg/dL (5.6 mmol/L), or previously diagnosed type 2 diabetes.

### Statistical analysis

Categorical variables were presented as frequencies and percentages, and the Chi-square test was used to test for dependencies between groups. Continuous variables were presented as means and standard deviations (SD), and Student’s *t*-test for independent samples was used to evaluate mean differences between normally distributed variables, whereas the Mann-Whitney U-test was used for non-normally distributed continuous variables. Normality was evaluated using the Kolmogorov-Smirnov test. Multiple logistic regression analyses were performed in order to identify factors independently associated with MetS. Further, multiple logistic regression analyses were used to assess the odds ratios (ORs) and respective 95% confidence intervals (CIs) of the association between quantity of coffee (espresso) and tea consumed and specific components of MetS, adjusting for gender, age, energy intake, and adherence to the MD. Analysis of contrasts was finally used to test trends among groups. Moreover, to evaluate the potential effect of the MD on the relationship between the caffeinated beverages and MetS, the median cut-off point of 28 for the MedDietScore was used in a supplemental logistic regression model to stratify the population studied into those more adherent and those less adherent to the diet. All reported *P*-values were based on two-sided tests and compared to a significance level of 5%. SPSS 17 (SPSS Inc., Chicago, IL, USA) software was used for all statistical calculations.

## RESULTS

Demographics of the 1889 subjects included in the survey evaluated with relation to MetS are presented in Table 1. The mean age of the study group was 50.2 years (SD 16.3 years) and almost 60% were female. About one third and almost the half of the population study consumed daily tea and coffee, respectively. Espresso was the most commonly consumed beverage containing caffeine. Subjects with increased age and BMI, low educational level, current smoking, low physical activity, low MedDietScore, low caffeine intake, high total energy intake, and not drinking coffee or tea were more likely to have MetS in general. In contrast, other types of beverages containing caffeine were not associated with MetS. The prevalence of the specific components of MetS by gender is reported in Table 2. Females had significantly increased WC and suffered more hypertension compared to males, who were more likely to have decreased HDL-cholesterol and increased fasting plasma glucose levels.

**Table 2.** Specific components of metabolic syndrome by gender

	Total (*n* = 1889)	Male	Female	*P*
WC ≥90 cm in men and ≥80 cm in women, *n* (%)	278 (14.7)	85 (11.2)	193 (17.1)	<0.001
SBP ≥130 mm Hg or DBP ≥85 mm Hg or hypertensive treatment, *n* (%)	1064 (56.3)	406 (53.4)	658 (58.3)	<0.05
HDL-c <40 mg/dL in men and <50 mg/dL in women, *n* (%)	264 (14.0)	123 (16.2)	141 (12.5)	<0.05
TG ≥150 mg/dL, *n* (%)	226 (12.0)	100 (13.2)	126 (11.2)	0.19
FPG ≥100 mg/dL or diabetes treatment, *n* (%)	432 (22.9)	202 (26.6)	230 (20.4)	<0.05

DBP, diastolic blood pressure; FPG, fasting plasma glucose; HDL-c, high-density lipoprotein cholesterol; SE, standard error; SBP, systolic blood pressure; TG, triglycerides; WC, waist circumference.

The variables associated with increased (or decreased) odds of having MetS were evaluated with univariate and multiple logistic regression models (Table 3). After adjusting for all covariates, age, physical activity, current smoking, MedDietScore, and coffee or tea consumption were associated with MetS, whereas caffeine consumption had no association.

**Table 3.** Univariate and multivariate analysis of factors associated with metabolic syndrome

	Metabolic syndrome			

	Crude	*P*	Multivariate^a^	*P*
	
	OR (95% CI)		OR (95% CI)	
Gender				
Male	1		—	
Female	0.99 (0.75–1.33)	0.99	1.26 (0.9–1.74)	0.16
Age, years (per 1 year)	1.02 (1.01–1.03)	<0.001	1.01 (1.00–1.02)	<0.05
BMI (kg/m^2^)	1.31 (1.26–1.36)	<0.001	1.28 (1.23–1.34)	<0.001
Educational level				
Low	1		1	
Medium	0.72 (0.54–0.95)	<0.05	0.81 (0.59–1.11)	0.18
High	0.36 (0.18–0.73)	<0.05	0.48 (0.23–1.00)	0.05
Socio-economic status				
Low	1		1	
Medium	1.03 (0.76–1.39)	0.84	1.00 (0.71–1.40)	0.99
High	0.83 (0.54–1.30)	0.42	0.95 (0.58–1.56)	0.88
Current smoking (yes)	1.60 (1.19–2.16)	<0.001	1.80 (1.29–2.51)	<0.001
Alcohol drinking (yes)	1.02 (0.77–1.37)	0.88	1.14 (0.82–1.59)	0.42
Physical activity level				
Low	1		1	
Moderate	1.07 (0.80–1.44)	0.63	1.06 (0.77–1.46)	0.72
High	0.33 (0.18–0.58)	<0.001	0.54 (0.29–0.99)	<0.05
MedDietScore				
Low	1		1	
Medium	0.58 (0.43–0.79)	<0.05	0.89 (0.63–1.26)	0.51
High	0.26 (0.14–0.48)	<0.001	0.50 (0.26–0.97)	<0.05
Caffeine (mg/day)				
<80 mg	1		1	
80–160 mg	0.56 (0.40–0.77)	<0.001	1.01 (0.64–1.58)	0.98
>160 mg	0.71 (0.49–1.02)	0.61	1.63 (0.92–2.89)	0.10
Source of caffeine				
Soft drinks	1.08 (0.82–1.43)	0.76	1.15 (0.74–1.56)	0.38
Tea	0.65 (0.48–0.90)	<0.05	0.51 (0.34–0.78)	<0.05
Energy drinks	0.83 (0.51–1.37)	0.40	0.86 (0.48–1.55)	0.62
Coffee	0.67 (0.50–0.90)	<0.05	0.43 (0.27–0.70)	<0.05

^a^Adjusted for gender, age, BMI, educational level, socio-economic status, energy intake, smoking status, alcohol drinking, physical activity level, MedDietScore, caffeine, and source of caffeine.

BMI, body mass index; CI, confidence interval; OR, odds ratio.

The association between coffee and tea consumption (mL/day) and the specific components of MetS was evaluated using multiple logistic regression analyses adjusted for age, gender, and adherence to the MD (Table 4). Triglycerides ≥150 mg/dL and fasting plasma glucose ≥100 mg/dL or diabetes treatment were significantly associated with both medium (45–90 mL/day) and high (>90 mL/day) intake of espresso coffee (only fasting plasma) and with medium (125–250 mL/day) intake of tea. When the analysis was repeated including smoking habit and physical activity level in the model, only blood triglycerides were still associated with both coffee and tea intake (Table 4).

**Table 4.** Multiple regression analyses for association between coffee and tea quantities consumption (mL/day) and specific components of metabolic syndrome

	Coffee consumption (mL/day)				Tea consumption (mL/day)			
	
	0	45–90	>90	*P* for trend	0	125–250	>250	*P* for trend
WC ≥90 cm in men and ≥80 cm in women								
Crude OR (95% CI)	1	0.91 (0.68–1.20)	1.24 (0.82–1.86)	—	1	0.74 (0.55–0.99)*	0.67 (0.28–1.59)	—
Adjusted OR (95% CI)^a^	1	0.87 (0.65–1.16)	1.38 (0.91–2.10)	0.12	1	0.76 (0.56–1.02)	0.71 (0.29–1.70)	0.06
Adjusted OR (95% CI)^b^	1	0.84 (0.63–1.13)	1.23 (0.80–1.89)	0.15	1	0.74 (0.55–1.01)	0.66 (0.27–1.59)	0.06
SBP ≥130 mm Hg or DBP ≥85 mm Hg or hypertensive treatment								
Crude OR (95% CI)	1	0.91 (0.75–1.11)	1.22 (0.89–1.70)	—	1	0.96 (0.78–1.17)	0.92 (0.53–1.60)	—
Adjusted OR (95% CI)^a^	1	0.89 (0.73–1.09)	1.29 (0.94–1.77)	0.27	1	0.98 (0.80–1.20)	0.94 (0.54–1.64)	0.06
Adjusted OR (95% CI)^b^	1	0.88 (0.72–1.08)	1.22 (0.88–1.68)	0.26	1	0.98 (0.80–1.20)	0.93 (0.53–1.62)	0.06
HDL-c <40 mg/dL in men and <50 mg/dL in women								
Crude OR (95% CI)	1	0.79 (0.59–1.06)	1.45 (0.96–2.12)	—	1	0.88 (0.66–1.18)	0.90 (0.40–2.02)	—
Adjusted OR (95% CI)^a^	1	0.78 (0.58–1.05)	1.47 (0.98–2.20)	0.23	1	0.89 (0.66–1.19)	0.90 (0.40–2.04)	0.10
Adjusted OR (95% CI)^b^	1	0.77 (0.57–1.04)	1.44 (0.96–2.15)	0.22	1	0.89 (0.66–1.19)	0.87 (0.39–1.97)	0.08
TG ≥150 mg/dL								
Crude OR (95% CI)	1	0.73 (0.53–1.01)	1.11 (0.71–1.73)	—	1	0.68 (0.49–0.95)*	1.19 (0.55–2.56)	—
Adjusted OR (95% CI)^a^	1	0.72 (0.52–0.99)*	1.19 (0.76–1.87)	0.07	1	0.71 (0.51–0.99)*	1.22 (0.56–2.64)	0.09
Adjusted OR (95% CI)^b^	1	0.71 (0.52–0.98)*	1.06 (0.67–1.68)	0.06	1	0.70 (0.50–0.97)*	1.17 (0.54–2.55)	0.08
FPG ≥100 mg/dL or diabetes treatment								
Crude OR (95% CI)	1	0.81 (0.64–1.02)	0.60 (0.40–0.90)*	—	1	0.71 (0.55–0.90)*	0.62 (0.30–1.28)	—
Adjusted OR (95% CI)^a^	1	0.75 (0.59–0.96)*	0.67 (0.44–0.94)*	<0.001	1	0.74 (0.58–0.96)*	0.67 (0.32–1.42)	<0.05
Adjusted OR (95% CI)^b^	1	0.84 (0.64–1.10)	0.71 (0.45–1.12)	<0.05	1	0.77 (0.59–1.02)	0.59 (0.25–1.41)	0.06

^a^Analysis adjusted for age, gender, energy intake, and adherence to the Mediterranean diet (MedDietScore).

^b^Analysis adjusted for age, gender, energy intake, adherence to the Mediterranean diet (MedDietScore), smoking habit and physical activity.

*P < 0.05.

CI, confidence interval; DBP, diastolic blood pressure; FPG, fasting plasma glucose; HDL-c, high-density lipoprotein cholesterol; OR, odds ratio; SE, standard error; SBP, systolic blood pressure; TG, triglycerides; WC, waist circumference.

Since adherence to MD was reported to be associated with MetS, we evaluated the effect of coffee and tea intake by stratifying the sample into high and low adherence to the diet (using a cut-off point of 28 on the MedDietScore). Among individuals more adherent to the MD, only tea was significantly associated with reduced odds of MetS (OR 0.37, 95% CI 0.16–0.86). In contrast, among those less adherent to the MD, both tea (OR 0.42, 95% CI 0.24–0.72) and coffee (OR 0.23, 95% CI 0.12–0.47) were associated with reduced odds of MetS. Regarding the analysis of single components of MetS, no associations with either coffee or tea consumption (mL/day) were found among subjects with high adherence, whereas a significant association with blood triglycerides was reported among those with low adherence to the MD (data not shown).

## DISCUSSION

In the present study, we found that intake of coffee and tea was associated with a significant reduction in the number of components of MetS and reduced prevalence of MetS. Despite the fact that increased caffeine intake was negatively associated with MetS, only the type of beverage (namely coffee and tea) was significant in the multivariate model. In contrast, caffeine intake neared significance for increased odds of having MetS, probably because the increased caffeine intake was associated with energy and soft drinks consumption. When analysis was conducted by stratifying the sample into high and low adherence to the MD, the association of coffee and tea consumption with MetS remained significant for more adherent individuals, whereas only tea still demonstrated an association among more adherent participants.

In our population, the prevalence of MetS was 11.9% (226/1889), slightly lower than the 16%–23% reported in Italy.^3^^,^^4^ As shown in Table 3, coffee and tea intake was inversely associated with MetS, indicating low levels of consumption in the population suffering from this cluster of risk factors. The components of MetS that have been found to be more susceptible to the protective effect of coffee and tea consumption were triglycerides and fasting plasma glucose, although the association with fasting plasma lost significance when lifestyle habits (ie, smoking and physical activity level) were considered in the multivariate model. It is conceivable that, in this last case, tea and coffee consumption were related to healthier lifestyle habits and, consequently, to better fasting blood glucose levels.

Although the exact mechanism through which coffee and tea may protect from cardiovascular risk factors is still unclear, it has been suggested that the antioxidant properties of polyphenols contained in coffee and tea may be involved. The main polyphenols in coffee are isomers of chlorogenic acid, whereas most of the polyphenols contained in tea belong to the catechin chemical family.^21^ All types of polyphenols are strong antioxidants and may prevent tissue damage caused by free radicals.^22^^–^^25^ Results of other epidemiological studies regarding the association between coffee intake and MetS are conflicting. Although some studies have found an inverse association,^13^^,^^14^^,^^26^ others reported no significant relationship^15^ between coffee intake and MetS. Epidemiological research on tea consumption has found more striking evidence of the favorable effects of tea intake on MetS, reporting that subjects consuming multiple cups per day were less likely to have MetS.^27^^–^^29^

However, a possible confounding factor may be the role of the overall diet quality of the population studied. Indeed, diet quality was often not taken into account in the previous studies of coffee and tea consumption. Studies reporting a beneficial effect were conducted in countries with well-known healthy dietary habits (ie, Japan),^30^ whereas those studies reporting inconclusive results were conducted in central and northern Europe, with no data on dietary habits given. In our study, we reported the association between MetS and the traditional Mediterranean dietary pattern, which shares several characteristics with the Japanese traditional diet (ie, low intake of red meat, high intake of fish, etc.).^31^ Subjects more adherent to the MD were less likely to have MetS or its components, as supported by many previous studies.^32^ According to our results, increased adherence to the MD was more strongly associated with MetS than coffee and tea consumption, whereas coffee and tea consumption had a positive impact mostly among low adherent individuals. We hypothesize that the health effects of such beverages are more evident in individuals with unhealthy (or rather non-healthy) dietary habits.

The current study has some limitations. First, methodological issues must be addressed. Since the study group was selected according to the availability of blood samples, our population was a convenient sample drawn from the original random sample. Further, blood sampling was not centralized, and information was retrieved retrospectively. Due to the cross-sectional nature of the study, a cause and effect relationship could not be elucidated. Other limitations related to food data must be reported. Information on additional sources of caffeine, such as dietary supplements, was lacking and not considered in the analysis. In addition, we were unable to differentiate types of tea consumed (ie, green or black). Finally, dietary information was self-reported, which may have led to recall bias. Despite such limitations, we believe our findings remain of significant value, since few studies have been conducted on this topic and no previous data regarding coffee and tea consumption in an Italian population have been published.

In conclusion, coffee and tea consumption was significantly associated with MetS and its components. Adherence to the MD was also associated with reduced odds of developing MetS. Since findings of epidemiological studies on the beneficial effects of coffee and tea on MetS are still mixed and puzzling, future research should investigate with a more holistic approach, taking into account potential confounding factors such as other dietary habits.
